# Effect of Basic Fibroblast Growth Factor on Achilles Tendon Healing in Rabbit

**Published:** 2017-01

**Authors:** Arash Najafbeygi, Mohammad Javad Fatemi, Amir Hussein Lebaschi, Seyed Jaber Mousavi, Seyed Abouzar Husseini, Mitra Niazi

**Affiliations:** Department of Plastic Surgery, Burn Research Center, Hazrate Fatemeh Hospital, Tehran University of Medical Sciences, Tehran, Iran

**Keywords:** Tendon injury, Healing, Fibroblast growth factor, Rabbit

## Abstract

**BACKGROUND:**

Tendon injuries are common and it takes a long time for an injured tendon to heal. Adverse phenomena such as adhesion and rupture are associated with these injuries. Finding a method to reduce the time required for healing which improves the final outcome, will lead to decreased frequency and intensity of adverse consequences. This study was designed to investigate the effects of basic fibroblast growth factor on the healing of the Achilles tendon in rabbits

**METHODS:**

In 10 New Zealand white rabbits, Achilles tendon was cut at the intersection of the distal and middle thirds on both hind legs. One microgram of recombinant basic fibroblast growth factor (bFGF) was injected in the proximal and distal stumps of the cut tendon on the right side (study group). Normal saline of equal volume was injected on the left side in the same way (control group). Then the tendons were repaired with 5/0 nylon using modified Kessler technique. A cast was made to immobilize each leg. On day 42, rabbits were euthanized and both hind legs were amputated. Tensometry and histopathologic examination were done on specimens.

**RESULTS:**

In tensometric studies, more force was required to rupture the repair site in study group. In histopathologic examination, collagen fibers had significantly better orientation and organization in the study group. No difference was noted regarding number of fibroblast and fibrocytes, and degree of angiogenesis in the two groups.

**CONCLUSION:**

Application of basic fibroblast growth factor at tendon repair site improves the healing process through improvement of collagen fiber orientation and increase in biomechanical resistance.

## INTRODUCTION

Tendon and ligamentous injuries are reported to consist approximately 50% of all musculoskeletal injuries.^1^ Tendon repair has been a challenging issue for surgeons in treating. Researchers have been looking for ways to improve tendon repair quality, reduce healing time and immobilization period, and expedite return to daily activities.^1^^,^^2^ Improvements in surgical techniques for repair, earlier active and passive exercises and rehabilitation are among efforts to achieve this end. Topical application of agents, such as growth hormone, methotrexate, honey, NSAIDs and steroids, PRP, and growth factors at repair site has recently been a focus of interest among researchers.^3^^,^^4^

Principle elements of tendons are collagen fibers (70%), cells, and extracellular matrix rich in proteoglycans. Collagen confers tensile strength to tendons. The ground matrix maintains the collagen fibers and their mature extracellular arrangements. Proteoglycans regulate tissue strength through controlling size and organization of collagen fibers.^5^ A large number of substances are influential in the tendon healing process. Of these, a few have been the focus of attention: insulin-like growth factor 1 (IGF-1), platelet-derived growth factor (PDGF), vascular endothelial growth factor (VEGF), transforming growth factor beta (TGF-β), and basic fibroblast growth factor (bFGF).^5^^,^^6^

IGF-1 exerts its effects through increase in collagen and proteoglycans during the inflammatory phase. PDGF is expressed in the early post-injury phases and increases growth factor synthesis and DNA proliferation in tenocytes. VEGF is effective in stimulating angiogenesis and increasing endothelial cell proliferation and cell migration and increases collagen deposition at tendon injury site.^5^^,^^6^ The three TGF-β subtypes TGF-β1, TGF-β2, and TGF-β3 have well-known effects in wound repair and scar formation. Bone morphogenic proteins are a subgroup of TGF-β, and are capable of enhancing bone and cartilage formation, tissue differentiation and fibrogenesis.^6^

Role of bFGF is still under active research. The beneficial effects of bFGF on the proliferation and collagen production of the tenocytes, differentiation of bone marrow stromal cells into osteoblasts, local anabolic effects in bones, angiogenesis, regeneration of neurons in the central and peripheral nervous systems, and enhancing the healing processes of the corneal epithelium have previously been reported.^6^ Therefore, the present study was designed to investigate the effects of bFGF on rabbit Achilles tendon repair.

## MATERIALS AND METHODS

Ten skeletally matured male White New Zealand rabbits of 10 to 14 months of age, and 2000-2500 g body weight were randomly divided into two equal injured-treated and injured-control groups. The study was approved by ethical committee of the university, and the care and surgery of the rabbits were performed in compliance with the international principles of animal use in research. 

The rabbits were anesthetized using ketamine of 50 mg/kg and xylasine of 5 mg/kg. Under sterile condition, an incision was made on the posterior aspect of each hind leg at the intersection of the proximal two thirds and the distal third. The Achilles tendon was identified and cut completely at the intersection of the middle and distal thirds. Human recombinant bFGF was diluted with normal saline, giving it the same viscosity as the normal isotonic saline. 

Animals were randomly divided into 2 groups for injections. In the first group, doses of 0.5 μg were injected subcutaneously at the proximal and distal stumps. Then the tendon was repaired by 5/0 nylon using the modified Kessler technique plus epitendinal sutures. The skin was repaired with separate simple 5/0 nylon sutures. The same procedure was done on the left side except that 0.5 ml of normal saline was used and 0.25 ml was injected into each proximal and distal stump as placebo. 

In the end, aluminum cast was used to immobilize the legs. Two rabbits died on days 10 and 12 due to unknown cause(s). The 8 remaining rabbits were euthanized using CO_2_ gas and the hind legs were amputated. In 5 rabbits (10 Achilles tendons), the force required to induce rupture at the repair site was measured through tensometry (SANTA, STM-20, Iran, load: 20 kg or 200 N, Speed: 5 mm/min). 

In 3 rabbits (6 Achilles tendons), the tendons were excised completely and were fixed in 4% formaldehyde for 5 days, processed, embedded in paraffin and sectioned in 3-μm thickness slices and stained using standard H&E staining. We used high power filed light microscopy (Zeiss, Germany) and a 3 grade scoring system. The number of fibroblasts, fibrocytes, vessels (indicative of angiogenesis), and collagen orientation were qualitatively classified as low (1), intermediate (2), and high (3). The analysis of data was done using SPSS software (version 16, Chicago, IL, USA). Kolmogorov test was used to examine normality of distribution. Man Whitney U test and t-test were used to compare data. Significance level was set to be 0.05.

## RESULTS

**Fig. 1:**
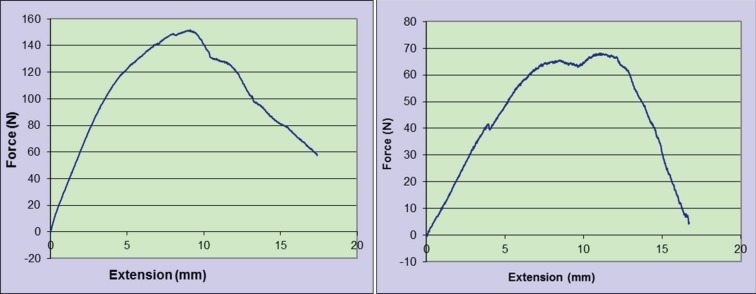
Fourth rabbit right and left tendon Tensiometrical digram

In the tensometric study, the mean and standard deviation of the forces to tear the stumps apart at the repair sites in study and control groups were 141.46±23.54 N and 92.12±21.23 N, respectively (*p*=0.008) (Table 1, Figure 1). The histopathologic findings were summarized in Table 2. The number of samples with intermediate and high number of fibroblasts in the study group were 5 (55.6%) and 4 (44.4%), respectively. Number of samples with intermediate and high number of fibroblasts in the control group were 8 (88.9%) and 1 (11.1%), respectively. Although number of fibroblasts appeared better in the study group, this difference was not statistically significant (*p*=0.12).

**Table 1:** Average tensiometry Force to tear the tendons in the control group and the group treated with fibroblast growth factor

**group**	**N**	**Mean**	**Std.Deviation**	**Minimum**	**Maximum**	***p*** **-value**
Beta Growth Factor	5	141.46	23.54	118.09	176.70	0.008
Control	5	92.12	21.23	68.30	115.63	
Total	10	116.79	33.51	68.30	176.70	

**Table 2:** Histopathologic Finding in the control group and the group treated with fibroblast growth factor

**Histopathologic variables**	**Group**			**Frequency**	**Percent**	***p*** **-value**
Fibroblast	Growth Factor	Valid	Moderate	5	55.6	0.12
			Severe	4	44.4	
			Total	9	100.0	
	Control		Moderate	8	88.9	
			Severe	1	11.1	
			Total	9	100.0	
Fibrocyte	Growth Factor	Valid	Moderate	4	44.4	0.33
			Severe	5	55.6	
			Total	9	100.0	
	Control		Moderate	2	22.2	
			Severe	7	77.6	
			Total	9	100.0	
Angiogenesis	Growth Factor	Valid	Moderate	3	33.3	0.36
			Severe	6	66.7	
			Total	9	100.0	
	Control		Moderate	5	55.6	
			Severe	4	44.4	
			Total	9	100.0	
Collagen Orientation	Growth Factor	Valid	Moderate	9	100.0	0.01
	Control		Moderate	5	55.6	
			Severe	4	44.4	
			Total	9	100.0	

Also, in histopathologic examination, number of samples with intermediate and high number of fibrocytes in the study group were 4 (44.4%) and 5 (55.6%), respectively. Number of samples with intermediate and high number of fibrocytes in the control group were 2 (22.2%) and 7 (77.8%), respectively. This difference was not statistically significant either (*p*=0.33). Considering angiogenesis, number of samples with intermediate and high number of vessels in the study group were 3 (33.3%) and 6 (66.7%), respectively. 

**Fig. 2:**
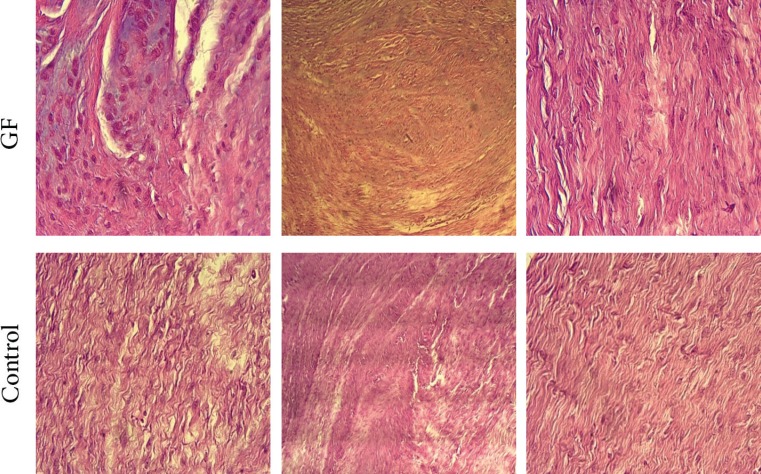
Histopathologic findings: number of fibroblasts and fibrocytes as well as Angiogenesis appeared better in the study group, but this difference was not statistically significant. Collagen orientation was moderate in all cases of the study group and a significant difference compared to the control group

Number of samples with intermediate and high number of vessels in the control group were 5 (55.6%) and 4 (44.4%), respectively. This difference was not statistically significant (*p*=0.36). Collagen orientation was moderate in all cases of the study group. In the control group, it was low in 5 (55.6%) cases and moderate in 4 (44.4%) cases (*p*=0.01) (Figure 2).

## DISCUSSION

We found that topical application of bFGF at the Achilles tendon repair site could possibly improve the healing process through enhancing collagen orientation and increasing biomechanical resistance. Fibroblast growth factors, or FGFs, are a family of growth factors, with members involved in angiogenesis, wound healing, embryonic development and various endocrine signaling pathways. In humans, 22 members of the FGF family have been identified, all of which are structurally related signaling molecules.^7^^-^^9^

FGF1 and FGF2 are more potent angiogenic factors than vascular endothelial growth factor (VEGF) or platelet-derived growth factor (PDGF).^10^ FGF1 has been shown in clinical experimental studies to induce angiogenesis in the heart.^11^ As well as stimulating blood vessel growth, FGFs are important players in wound healing. FGF1 and FGF2 stimulate angiogenesis and the proliferation of fibroblasts that give rise to granulation tissue. Regarding the role of bFGF in tendon repair, the earlier works in the last years of the twentieth century indicated the increased expression of this cytokine in tendon injury environment in canine and rabbit models.^12^^,^^13^

The influence of bFGF on the structural organization of the tendon including improved tissue alignment and crimp formation, enhanced cell maturation, increased collagen fibrils differentiation and maturation with decreased peritendinous adhesion are possibly the most significant effects of bFGF on the tendons healing. Enhanced percentage dry weight and collagen density are the consequences of these structural changes.^13^ Treatment with bFGF possibly relieved acute inflammation and post-surgical edema and resulted in painless movement. It has been shown that bFGF inhibited acute inflammation and pain by reducing the prostaglandine E2 concentration, nitric oxide activity and scavenging oxygen free radicals.^6^

Expression of bFGF in the inflammatory response present at the repair site has also been examined. Harwood *et al.* demonstrated increased expression of α5β1 and α(v)β3 by tendon cells exposed to either bFGF or platelet-derived growth factor-BB over a wide range of growth factor concentrations employed in the study.^14^ Due to this increased expression and potential benefits of these growth factors, during the early years of the last decade, bFGF has been used alone or in combination with other growth factors in various methods to evaluate its role in the tendon repair process. Application of bFGF has been shown to promote cellular proliferation and collagen synthesis in vivo.^15^^,^^16^

In a canine model of flexor tendon repair, it was demonstrated that bFGF was present in inflammatory cells surrounding the repair site.^17^ bFGF used in combination with other growth factors has shown improved effect over isolated usage.^18^^-^^20^ An important issue regarding design of studies to evaluate in vivo effects of various growth factors, including bFGF, has been the method of delivery of the agents to the repair site. The majority of studies so far have comprised the local application of the growth factors at the time of repair. In an ingenious design, Hamada and colleagues evaluated the effect of bFGF-coated nylon suture on tendon repair and found that this technique could increase the biomechanical strength of tendon repair and the thickness of epitenon during a 3-week period.^21^

Subcutaneous and continuous infusion has also been among known administration methods. A novel method to introduce bFGF into the repair site has been the use of viral vectors.^21^ Adeno-associated virus-mediated gene transfer is promising in the delivery of genes to tendons because this vector stimulates few adverse tissue reactions. In a study by Tang and coworkers, it was demonstrated that basic fibroblast growth factor gene transfer to digital flexor tendons by means of adeno-associated viral vector-2 significantly increases healing strength during the critical tendon healing period but does not increase adhesion formation.^23^

 We used direct injection of growth factor into tendon ends before repair for benefit of high concentration at repair site. In the clinical setting of tendon repair, surgeons are primarily concerned with repair characteristics (mainly time required for acceptable repair and its strength) and adhesion formation. These two aspects of tendon repair have also been an important arena for investigating the role of various growth factors, including bFGF. Results of researches have not been conclusive though. 

Ide* et al.* investigated the effect of application of bFGF on the tendon-to-bone remodeling of repaired supraspinatus tendon in rats subjected to bilateral detachment. The initial tendon-to-bone remodeling was accelerated by a local application of this growth factor.^24^ In our study the orientation of collagen was significantly better in growth factor group which is in the same direction with Ide *et al.*’s study. Thomopoulos and colleagues evaluated the effects of exogenous bFGF on intrasynovial flexor tendon healing in a canine model. They used sustained delivery of bFGF, at two different doses, for the study group.^25^

Vascularity, cellularity, and adhesion formation were increased in the tendons that received basic fibroblast growth. Their results indicated that although bFGF accelerated the cell-proliferation phase of tendon healing, it also promoted neovascularization and inflammation in the earliest stages following the suturing of the tendon. Despite a substantial biologic response, the administration of basic fibroblast growth factor failed to produce improvements in either the mechanical or functional properties of the repair. Rather, increased cellular activity resulted in peritendinous scar formation and diminished range of motion. Due to these results it may be better to inject the growth factor directly into tendons, as we did, to prevent pretendinious adhesion.^24^^,^^25^

In another research, effect of local bFGF and 5-fluorouracil on accelerating healing and preventing tendon adhesion after flexor tendon repair was investigated in a chicken model. The authors concluded that local single-use bFGF and 5-FU can not only effectively promote the healing of flexor tendon, but also significantly reduce tendon adhesion.^26^ The result of this study is the same with promotion of healing but we did not study the pre-tendinious adhesion formation.

The effects of recombinant human bFGF on a complete superficial digital flexor tendon (SDFT) rupture after surgical repair in rabbits were previously demonstrated. Compared to injured control animals, treated animals showed a decrease in the diameter of the injured tendon and peritendinous adhesion as well as increased tenoblast proliferation, collagen production and ultimate strength of the injured tendons.^6^^,^^27^

Based on the present findings, by designing suitable times of administration and dosage and leaving enough time for structural and functional development, it is possible that bFGF might be one of the drugs of choice in improving the structural organization and biomechanical performance of the injured tendons. These beneficial effects are possibly related to the potential capacity of bFGF to inhibit peritendinous adhesion formation, shorten the inflammatory processes, enhance cellular maturation, improve collagen deposition and facilitate collagen fibrils aggregation and differentiation. 

Further biochemical and molecular studies are needed to elucidate other aspects of the mechanism of action of this reagent on the structural and functional performance of tendon injuries. There are many limitations in our study. First, the low number of animals and pathologic specimens, Second,using only one type of coloring for histopathological examination. Therefore, it is recommended that the study should be done more precisely and with a larger sample size.
